# Case Management for People with Acquired Brain Injury with Complex Problems (Part 1): Outcomes of a One-group Trial

**DOI:** 10.5334/ijic.8649

**Published:** 2025-07-07

**Authors:** Annemarie P. M. Stiekema, Bjorn Winkens, Desiree Bierlaagh, Mireille Donkervoort, Natska Jansen, Kitty H. M. Jurrius, Judith Zadoks, Caroline M. van Heugten

**Affiliations:** 1 Department of Psychiatry and Neuropsychology, School of Mental Health and Neuroscience, Maastricht University, Maastricht, The Netherlands; 2 Limburg Brain Injury Center, Maastricht University, Maastricht, The Netherlands; 3 Department of Methodology and Statistics, Care and Public Health Research Institute (CAPHRI), Maastricht University, Maastricht, The Netherlands; 4 Desiree Bierlaagh (self-employed), Houten, The Netherlands; 5 Health Care and Social Work Division, Windesheim University of Applied Sciences, Almere, The Netherlands; 6 Mevrouw Slimmer Werken Social Innovation in Health Care and Well-Being, Drogteropslagen, Netherlands; 7 Brain Injury Team, Overijssel, The Netherlands; 8 In-Tussen Foundation, Utrecht, the Netherlands; 9 BreinDok Innovation in Care, Utrecht, The Netherlands; 10 Department of Neuropsychology and Psychopharmacology, Faculty of Psychology and Neuroscience, Maastricht University, Maastricht, The Netherlands

**Keywords:** acquired brain injury, caregivers, integrated care, psychosocial, long-term, outcomes

## Abstract

**Introduction:**

Many people with acquired brain injury (PwABI) and their family face long-term psychosocial problems and unmet needs. Currently, there are no structural and integrated health care services supporting life after brain injury. We evaluated Case Management (CM) for PWABI which aims to facilitate access to and integration of health care and social services for people with complex problems.

**Methods:**

One-group repeated measures study including 62 PwABI and 36 caregivers in the Netherlands. Assessments were conducted every six months for 18–24 months. Primary outcome was psychosocial well-being (Hospital Anxiety and Depression Scale). Secondary outcomes were self-efficacy, participation, life satisfaction, and needs for PwABI and caregivers; and caregiver burden.

**Results:**

Anxiety reduced significantly in both PwABI and their caregivers. Over time, PwABI reported significantly fewer unmet needs, but more participation restrictions. Caregivers reported significantly less caregiver burden and more self-efficacy over time.

**Discussion:**

CM seems promising for reducing unmet needs in PwABI and improving some psychosocial outcomes in PwABI and caregivers. Lifelong CM may however be necessary. A randomized controlled study is needed to confirm whether the positive outcomes are due to CM.

**Conclusion:**

This study warrants further research to establish the effectiveness of CM for PWABI.

## Introduction

Many individuals with acquired brain injury (ABI) face physical and neuropsychological consequences that heavily impact their daily life and that of their family members [1,2,3]. Cognitive, emotional, and behavioural difficulties can impede return to work or school and affect social life [4,5]. Limitations in functional abilities require support in daily living, often provided by family members [2,6]. The effects of ABI may fluctuate over time and adaptation challenges for patients and caregivers may emerge or re-emerge [1]. People with ABI (PwABI) describe the recovery process as slow and continuous [1,7,8]. The lifelong and dynamic impact of ABI is increasingly recognized, and long-term support is important for improving independence and societal participation for PwABI and alleviating caregiver strain [2,3,6,9,10].

Specialized care for PwABI often does not extend past hospital or rehabilitation discharge, and support for family members is lacking. Outpatient care may involve providers who have insufficient knowledge of how the persisting consequences of brain injury give rise to psychosocial issues. Additionally, support for ancillary needs related to housing, transportation, finances, and return to work is often hard to find [9]. PwABI and their family members struggle to navigate health care systems and access appropriate services [6], which may result in negative outcomes such as unemployment and psychosocial issues [1]. To optimize community integration and lead a fulfilling life, a key coordinator who proactively integrates appropriate care services to address diverse needs according to collaboratively formulated long-term strategies is essential [6,11,12,13].

Within the field of dementia care, Case Management (CM) is developed to improve the quality of care by stimulating collaboration, communication and access to available resources [14]. Also in people with diabetes or cancer, CM shows promising results [15]. There is evidence to support that CM reduces neuropsychiatric symptoms and improves the quality of life of people with dementia [16]. Moreover, among other forms of integrated care, CM is likely to reduce costs and improve outcomes [17]. However, the evidence is limited and considerable variation is present in terms of definitions, models, components and tasks/activities of the case manager (CMr) [18]. A Brain Injury Case Management taxonomy (BICM-T) has been suggested to overcome this variation and to develop a common language within the field of PwABI [19]. This taxonomy was subsequently used in a content analysis study showing that communication about the roles and actions of CMr needs to be improved [20]. Despite recommendations from experts and patients, rigorous research on its effectiveness is still lacking [20,21]. The studies on case management for PWABI reviewed in Lannin et al [21] were all conducted before 2005, and – to the best of our knowledge – no new outcome studies have been published since. In a recent systematic review on integrated care pathways for people with Autism Spectrum Disorder, similar to ABI in its heterogeneity and complexity, it was suggested that resear ch priority should be given to the identification of an integrated care pathway ‘model’ centered around CM [22].

We therefore developed case management for PWABI in the Netherlands along the framework proposed by Lukersmith and colleagues [19] and descriptions of CM for people with dementia in the Netherlands [23]. The new intervention was delivered in two separate studies. A randomized controlled trial on ‘early’ CM (i.e., from hospital visit or admission onwards) compared to usual care (for design, see [23]). In this study, the benefits of early CM were not shown, but access to CM was hindered due to unsuccessful monitoring of PwABI after hospital discharge [23]. In this explorative uncontrolled trial, we evaluated the outcomes of ‘late’ CM for PwABI with complex problems affecting multiple life domains and their family. We expected CM to improve psychosocial well-being. This is most relevant for PwABI living in the community, as common outcomes of CM in a more medical context (e.g. glycemic control for diabetes, rehospitalization for cancer) or neurodegenerative diseases (e.g. institutionalization for dementia) do not apply. The primary outcome was emotional well-being of PwABI. Secondary outcomes were participation, quality of life, self-efficacy, and care needs of PwABI and family members, and caregiver burden. The process evaluation in which we assessed the feasibility of CM alongside the outcome evaluation is presented elsewhere.

## Methods

### Design

This is a repeated measures uncontrolled trial conducted between October 2019 and December 2022 in three regions of the Netherlands: Flevoland, Utrecht, and Overijssel. These three regions volunteered in hosting the pilot CM as part of the national project (see section on Case Mamagement). The study protocol was approved by the Ethics Review Committee Psychology and Neuroscience at Maastricht University (ERCPN-212_01_09_2019). All participants gave informed consent. We calculated the sample size based on a paired-samples t-test, a Cohen’s d (standardized effect size) of 0.37 (derived from a monitoring/psycho-educational intervention using the Hospital Anxiety Depression Scale (HADS) [24], an alpha of .05, power of 80%, and accounting for a drop-out rate of 30%. Our goal was to include 86 PwABI, with 60 completing the study.

### Participants

Adults with ABI and their loved one were included if the PwABI received CM due to experienced unaddressed problems due to a neurologically confirmed ABI, had sufficient command of the Dutch language and were willing and able to provide informed consent. During recruitment, it appeared that CMrs also provided care to people with comorbid neurodegenerative disease or without objectified brain injury. We decided to include those in the study as well. No exclusion criteria were held.

### Casemanagement intervention

The Dutch Ministry of Health, Welfare, and Sport funded a national pilot project that developed and evaluated CM for PwABI and their family. The aim of CM is to help PwABI and their family members regain control over their lives by facilitating access to and integration of healthcare and social services. The CMr, a designated contact person for PwABI and their families, works in a person-centered way and adapts to their needs. The CMr does not provide care but rather coordinates care provision, stimulates collaboration between health care professionals, organizations and administrative and fincancial parties. In addition, the CMr provides a listening ear, thinks along, gives individual information and advice. This can be seen as personalized psychosocial support. Contact between the CMr and the PwABI and their family was either by home visits, telephone or email conversations. Depending on the needs of the clients the support could vary from information provision, to filling in forms or calling an organization, or just listening. As soon as possible, the CMr hands over to regular care but remains available and in touch to address any new needs (for a detailed overview of CM actions and definitions, see [19,20]).

### Casemanagement pilot

Three CMr teams were formed for this pilot, one in each participating region. As the project progressed, cases were assigned to CMrs based on their expertise and the participants’ needs rather than their location. CMrs all have expertise in working with PwABI, but their professional backgrounds differ. The teams consisted of social workers, speech and language therapists, job coaches, nurses, and peer support workers. The CMrs underwent a 4-day training program and attended several half-day meetings to familiarize themselves with their role as CMr. During the pilot period, the project team leaders met regularly with CMrs to discuss common themes and to offer further training on topics they deemed relevant. There was no intervention protocol as the content, form and intensity of CM varies according to PwABI’s and family’s specific needs. Lukersmith et al. defined the following main elements of CM: engagement, holistic assessment, planning, education, training and skills development, emotional and motivational support, advising, coordination and monitoring [18,20]. We used this framework for evaluating actual CM activities and determine if any adjustments are necessary. The case management elements are presented in Table 1.

**Table 1:** Case management elements.


Monitoring	Tracking functioning and well-being of people with ABI and family members. In the present study, a digital monitoring system is used for this purpose (described below).

Identification	Identification of questions, problems and needs (based on monitoring) that hinder functioning and well-being at the time they emerge.

Assessment	Assessing the nature and severity of the presented problem, burden on and capabilities of the person with ABI and the family member, the role of their social network, making implicit or unmentioned questions and problems explicit, drawing conclusions about the core problem in the individual context

Information	Providing information and education on the (impact of) ABI to assist understanding, information or education related to the question or problem (with a focus on capabilities to self-manage the problems), informing on available care and support services.

Support	Guiding decision-making with regard to managing the problem, providing practical or psychosocial support for relatively mild problems (focused on maintaining or improving self-management).

Referral	Referring to more specialized care or support for relatively complex problems and guiding decision-making with regard to what available services to use.

Coordination	Supporting access to services, facilitating collaboration between different service providers and bringing about appropriate care when this is not available through the regular services.


CMrs were employed by an independent foundation created for this project and supervised by the project team leaders who operate independently from healthcare organizations or institutions. CMrs often worked in teams of two, for example with one CMr focusing on the family member while the other focused on the PwABI.

Participants for the pilot were recruited via the CMr teams. Professionals in the regions could refer patients to the project team, and patients presented themselves to the project team as well. This is an addition to care as usual. A member of the project team informed PwABI and if available, a family member, about the study. They also explained that CM would be provided regardless of participation in the study.

## Outcomes

### PwABI outcomes

The primary outcome is the total score of the Hospital Anxiety and Depression Scale (HADS [25]), assessing symptoms of anxiety and depression with 14 items scored on a 4-point scale ranging from 0 to 3 with varying anchors. Additionally, scores were calculated for the anxiety and depression subscales, where subscale scores of >7 indicate the presence of an anxiety disorder or depression. The psychometric quality of the scale is sufficient [26].

Secondary outcomes included measures for psychosocial outcomes, self-efficacy, and care needs. The Utrecht Scale for Evaluation of Rehabilitation-Participation (USER-P [27]) restriction subscale was used to assess restrictions in vocational, leisure and social activities as a consequence of ABI. The scale has nine items ranging from 0 (not possible) to 3 (without difficulty) and a ‘not applicable’ option. Scaled total scores with a possible range of 0–100 were calculated based on the applicable items; higher scores indicate less participation restrictions. The USER-P has shown sufficient reliability and validity [27].

The Life Satisfaction Questionnaire (LiSat [28]) measures satisfaction with life as a whole, self-care management, contacts with friends, work life, family life, partner relationships, financial situation, leisure, and sex life. Its nine items are scored on a 6-point scale (from 1 “very dissatisfied” to 6 “very satisfied”) with satisfactory reliability and validity [29,30].

Self-efficacy was measured with the Patient Activation Measure (PAM [31]). The PAM assesses self-reported knowledge, skills, and confidence for self-management of one’s health or chronic condition with 13 items scored on a scale of 0 to 4 (disagree strongly to agree strongly) and a not applicable option. An algorithm was used to transform the scores on the PAM to different levels of self-management, from ‘disengaged and overwhelmed’ to being their own health advocate. The PAM has shown good psychometric properties [32].

Care needs were measured with the Longer-term Unmet Needs after Stroke questionnaire (LUNS [12]). The LUNS includes 22 dichotomous (yes/no) items and one open ended question assessing needs regarding the physical, social, and emotional consequences of stroke. We changed the word ‘stroke’ into ‘brain injury’ in two items to make the scale applicable for all types of ABI. Scores range from 0 to 22, higher scores indicating more needs. The LUNS is reliable and valid [33].

### Caregiver outcomes

For family members, the primary outcome measure was also the HADS total score, which they completed for themselves. Secondary outcomes included assessments of family members’ psychosocial well-being, self-efficacy, and care needs. The LiSat was also administered with family members.

The Caregiver Strain Index (CSI [34]) was used to assess caregiver burden. The 13 items of the CSI can be rated with ‘yes’ or ‘no’ and total scores ranging from 0–13. Higher scores reflecting higher caregiver burden, a score of 7 or higher indicated substantial burden. The scale has shown sufficient validity and reliability [35].

*S*elf-efficacy regarding care management and service use was assessed using the Carer Self-Efficacy Scale (CSES; [36]). Its ten items were scored on a 10-point scale from ‘not at all certain’ to ‘very certain’; higher scores on the CSES indicate higher levels of self-efficacy. Reliability and validity of the scale are sufficient [37].

Caregiver needs were assessed with the Family Needs Questionnaire (FNQ; [38]). The scale includes 40 items assessing needs regarding health information (tange 0–20), emotional support (range 0–16), instrumental support (range 0–12), professional support (range 0–10), community support network (tange 0–10), and involvement with care (range 0–6). Caregivers indicated the importance of each perceived need and rated the degree to which the need was met. Higher scores indicate more needs. The FNQ has shown sufficient validity and reliability [39,40].

### Procedures

PwABI who received CM were eligible for participation in the study if they met all criteria. If the PwABI was interested, the CMr forwarded their contact details to the research team. The researcher then explained the procedures in full, collected written informed consent and planned the baseline assessment. After the baseline assessment, participants were provided with CM and they were able to utilize it for the entire duration of the study. Assessments took place every 6 months and were administered via digital questionnaires if possible, and on paper or via telephone based on the abilities and preference of the participants. Depending on the time of inclusion, participants were followed up for 18 or 24 months until the end of data collection in December 2022 (predefined study end date by the subsiding party).

### Statistical analysis

Demographic and injury-related characteristics were analysed descriptively. The longitudinal trends in numerical outcomes were assessed using linear mixed models (or marginal models for repeated measures), where the fixed part consisted of time (0, 6, 12, 18 months), gender (male, female), education level (low, middle, high) and age (in years). To account for the correlation between repeated measures from the same patient, several options for the covariance structure of the repeated measures (unstructured and the homogenous and heterogeneous version of first-order auto-regression (with moving average), Toeplitz and CS) were considered. In addition, random intercept and/or slope models (unstructured or variance components) were applied to the data as well as combinations of these random parts with an AR1 structure for repeated measures. The final model was based on the lowest Bayesian Information Criterion (BIC), while the models were also compared using a likelihood ratio test if possible (hierarchically nested). No multiple imputation was applied as the likelihood-based approach was used for missing outcome values. Restricted maximum likelihood estimates of the mean value at each time point as well as the mean differences with baseline are reported with the corresponding 95% confidence intervals (CI). As sensitivity analysis, the final model was also applied to the data including the last time point (24 months).

For PAM levels, an ordinal logistic mixed model was used with the same fixed part as for the aforementioned model, where a random intercept on patient level was included to account for the correlation between repeated measures.

Two-sided p-values ≤ 0.05 were considered statistically significant. A Bonferroni correction for three pairwise comparisons with baseline was used. All analyses were performed using IBM SPSS Statistics for Windows (version 28, Armonk, NY, USA).

## Results

### Participants

From October 2019 to June 2021, 239 patients were referred to or contacted the CM project team, of whom 90 were forwarded to the research team. Reasons for not referring patient to the researchers included limited cognitive/communicative abilities, lack of insight (CM was aimed at the partner) and questionnaire burden. Of the referred 90 PwABI, reasons for exclusion were questionnaire burden (n = 8), multiple contacts with a CMr before baseline assessment could be completed (n = 6), limited communicative/cognitive abilities (n = 3), specific personal circumstances (n = 5) and unknown (n = 6). The remaining 62 PwABI completed the baseline assessment, 36 PwABI had a family member participate. Demographic characteristics are shown in Table 2.

**Table 2:** Demographic and injury-related information of participants.


	PEOPLE WITH ABI (n = 62)	FAMILY (n = 36)

Age, years (mean ± standard deviation)	53.79 ± 15.08	56.67 ± 10.65

Gender, F/M	29/33	29/7

Educational level, %^a^		

Low (1–4)	19.4	13.9

Middle (5)	50.0	55.6

High (6–7)	30.6	30.6

Type of brain injury*, %		

Cerebrovascular accident	53.2	

Traumatic brain injury	27.4	

Transient ischaemic attack	9.7	

Hypoxia	3.2	

Brain tumour	3.2	

Other cerebrovascular disorder	1.6	

Meningitis	1.6	

Time since injury, years (mean ± standard deviation)	5.7 ± 7.74	

Prior brain injury, %	35.5	

Living situation, %		

Alone	26.2	

With partner	37.7	

With partner and children	24.6	

With children	4.9	

With other people	6.6	

Unknown	1.6	

Relationship with person with ABI, %		

Partner		66.7

Parent		16.7

Child		8.3

Sibling		8.3

Living together with person with ABI, yes/no		77.8


*Diagnosis was objectified by a medical specialist in 53 cases, of two this was unknown, of seven patients their inclusion was based on receiving care on a brain injury indication or complaints that suggest brain injury. ^a^ Based on Verhage educational categories.

### Casemanagement

On average over the 24 months period of assessment, the CMR offered 2.4 hours support per participant per month. Most support was delivered during the first 6 months (mean 5.0 hours with a peak in the second month) which declined over time (2.5 hours at 12 months, 1.2 at 18 months and 0.6 at 24 months). See supplementary Figure 1.

### Results people with ABI

#### Primary outcome

**Figure 1:**
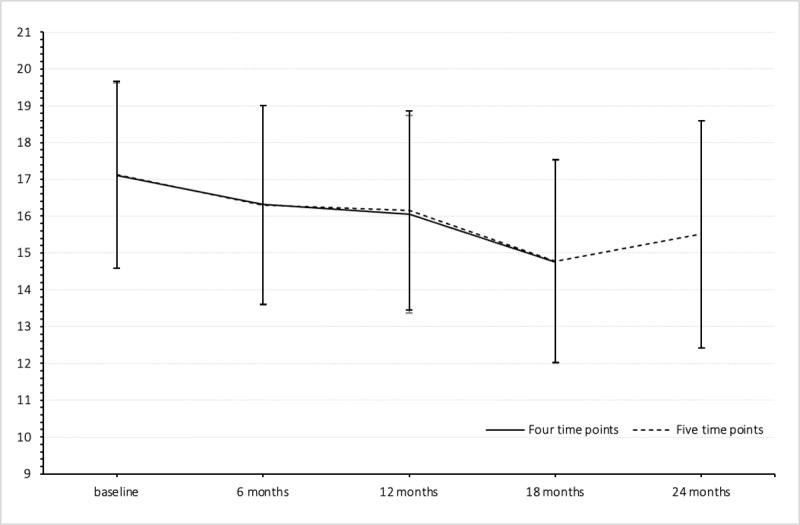
Estimated marginal means of total scores of the Hospital Anxiety and Depression Scale per time point of PwABI. Whiskers indicate 95% confidence intervals.

Table 3 shows results of the multilevel analyses of the HADS total scores. The overall tests revealed that there were no significant differences over time. Sensitivity analyses including the last time point showed similar results, and no significant difference between total scores at baseline and 24 months (data not shown; *p* = .220). Figure 1 shows the estimated marginal means of the total scores of the HADS per time point.

**Table 3:** Results for the Hospital Anxiety and Depression Scale total scores for PwABI.


	ADJUSTED EFFECTS ESTIMATES				

	N	OBSERVED MEAN (SD)	MEAN DIFFERENCE (95% CI)	P-VALUE	OVERALL

baseline	62	17.37 (8.87)	reference		F(3) = 1.963, p = .123

6 months	43	16.33 (8.80)	–0.79 (–2.63–1.05)	.397	

12 months	44	15.98 (9.21)	–1.06 (–2.88–0.77)	.253	

18 months	38	14.80 (9.39)	–2.34 (–4.27 – –0.42)	.017*	


*No longer significant after applying Bonferroni correction.

#### Secondary outcomes

The results of the multilevel analyses of the secondary outcomes can be found in Supplementary Table 1. The results of ordinal logistic mixed model analysis on PAM level are displayed in Supplementary Table 2. There were no significant time effects for the HADS depression subscale, LiSat, PAM scores and PAM level.

There was a significant overall time effect for HADS anxiety subscale (F(3) = 2.772, *p* = .044). Anxiety scores showed a significant reduction (fewer anxiety symptoms) between baseline and 12 months (mean difference: –1.23, 95% CI: –2.37 – –0.08), but this was no longer significant after Bonferroni correction. The reduction from baseline to 18 months was significant (mean difference: –1.616, 95% CI: –2.82 – –0.41), also after Bonferroni correction. Sensitivity analyses including the last time point showed similar results for the pairwise comparisons, but the overall time effect was no longer significant (data not shown; F(4) = 2.248, *p* = .067).

Furthermore, there was a significant overall time effect for USER-P restrictions subscale scores (F (3) = 5.527, *p* = .001). Significant differences (more restrictions) were found between baseline and 6 months (mean difference: –7.52, 95% CI: –11.66 – –3.39), baseline to 12 months (mean difference: –10.00, 95% CI: –15.49 – –4.52) and baseline to 18 months (mean difference: –6.93, 95% CI: –13.49 – –0.38). The difference between baseline and 18 months was no longer significant after Bonferroni correction. Sensitivity analyses including the last time point showed similar results for the pairwise comparisons, but the difference between baseline and 24 months was not significant (*p* = .193).

In addition, there was an overall time effect for LUNS scores over time (F(3) = 15.059, p < .001). All comparisons with baseline were significant (fewer unmet needs): 6 months (mean difference: –1.62, 95% CI: –2.64 – –0.60), 12 months (mean difference: –2.41, 95% CI: –3.44 – –1.37) and 18 months (–3.39, 95% CI: –4.44 – –2.34). Sensitivity analyses including the last time point showed similar results for the pairwise comparisons and a significant difference between baseline and 24 months (mean difference: –3.12, 95% CI: –4.38 – –1.86).

### Results for family members

#### Primary outcome

**Figure 2:**
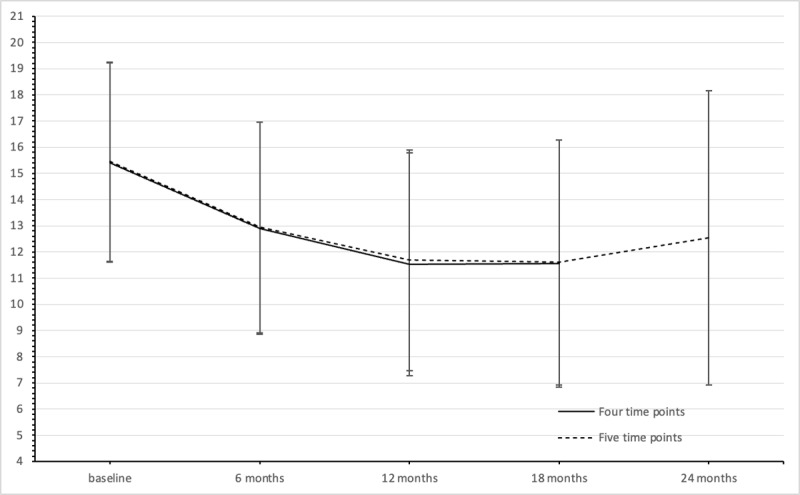
Estimated marginal means of total scores of the Hospital Anxiety and Depression Scale per time point of family members. Whiskers indicate 95% confidence intervals.

Table 4 shows results of the multilevel analyses of the HADS total scores. Comparisons between baseline and 12 months, and baseline and 18 months showed significant reductions over time, but the latter was no longer significant after Bonferroni correction. Moreover, the overall tests indicated no significant differences over time. Sensitivity analyses including the last time point showed similar results, and no significant difference between total scores at baseline and 24 months (data not shown; F(4) = 2.078, *p* = .098). Figure 2 shows the estimated marginal means of the total scores of the HADS per time point.

**Table 4:** Results for the Hospital Anxiety and Depression Scale total scores for family members.


	ADJUSTED EFFECTS ESTIMATES				

	N	OBSERVED MEAN (SD)	MEAN DIFFERENCE (95% CI)	P-VALUE	OVERALL

baseline	36	16.78 (8.60)	reference		F(3) = 2.639, *p* = .060

6 months	25	14.12 (8.70)	–2.51 (–5.09–0.06)	.055	

12 months	24	12.92 (8.25)	–3.88 (–6.81 – –0.96)	**.010**	

18 months	21	13.62 (9.21)	–3.86 (–7.46 – –0.25)	.037*	


*No longer significant after applying Bonferroni correction.

#### Secondary outcomes

The results of the linear mixed model analyses of the secondary outcomes can be found in Supplementary Table 3. There were no significant time effects for the HADS depression subscale, LiSat, CSES care management, FNQ emotional support, FNQ health information, FNQ instrumental support, FNQ involvement with care and FNQ professional support.

There was a significant overall time effect for HADS anxiety subscale (F(3) = 4.823, *p* = .004). Anxiety scores showed a significant reduction (fewer anxiety symptoms) between baseline and 12 months (mean difference: –2.50, 95% CI: –4.22 – –0.96) and baseline to 18 months (mean difference: –2.75, 95% CI: –4.46 – –1.04). Sensitivity analyses including the last time point showed similar results, but the difference between baseline and 24 months was not significant (*p* = .186).

Furthermore, there was a significant overall time effect for CSES care use scores (F(3) = 3.103, *p* = .032). Significant differences (more self-efficacy) were found between baseline and 6 months (mean difference: 3.29, 95% CI: 1.04–5.55) and baseline to 12 months (mean difference: 3.36, 95% CI: 0.51–6.20). The difference between baseline and 12 months was no longer significant after Bonferroni correction. Sensitivity analyses including the last time point showed similar results, and a significant difference between baseline and 24 months (mean difference: 5.92, 95% CI: 2.01–9.84).

In addition, there was a significant overall time effect for CSI scores (F(3) = 4.464, *p* = .006). Significant differences (lower caregiver burden) were found between baseline and 12 months (mean difference: –1.15, 95% CI: –1.10–0.19), and baseline and 18 months (mean difference: –0.80, 95% CI: –1.49 – –0.12). The difference between baseline and 18 months was no longer significant after Bonferroni correction. Sensitivity analyses including the last time point showed similar results, and a significant difference between baseline and 24 months (mean difference: 1.65; 95% CI: –2.52 – –0.77).

While no significant time effect was found for FNQ emotional support on the analysis with four time points, the overall time effect was significant for the analysis including the last time point (F(4) = 2.669, *p* = .039). A significant difference (more emotional support) was found between baseline and 24 months (mean difference: 3.59, 95% CI: 0.73–5.57), but this was no longer significant after Bonferroni correction.

## Discussion

This study examined psychosocial outcomes in people with complex problems after ABI and their family receiving CM, an integrated care service facilitating access to and integration of health, social and other services. The findings of the study demonstrated that individuals with ABI who received CM had a reduction in the number of unmet needs, accompanied by a decrease in anxiety symptoms but not with improvements in participation or life satisfaction. Family members did not experience a decrease in care needs, but showed a reduction in anxiety symptoms over time. Additionally, self-efficacy regarding care use improved after six months and caregiver burden was alleviated after 12 months, but these effects were not maintained in the long term.

We developed CM for PwABI according to the elements suggested by Lukersmith [18,20]. We can, however, not compare our findings to other studies employing this framework because these are not conducted yet. In the systematic review on CM for PwABI [21] the authors concluded that there was limited evidence that CM increased contact with community services and improved vocational outcomes. We did not measure contact with services, but our outcome that unmet needs reduced may be a consequence of more contact. Vocational outcomes were not improved in our study as shown by the results on the USER-P. In comparison to the outcome of CM in other fields, improvements in quality of life were also sparsely found in CM for diabetes [41] and only in the short term for dementia [16]. Nurse-led CM in cancer care did show effectiveness in quality of life, but these programs were already initiatied during hospital admission and were more intensive [42].

Despite some positive changes in outcomes over time, the general pattern of outcomes reflects that this group is severely affected, with problems in multiple life domains. Both PwABI and family members continued to experience high levels of psychosocial distress, as evidenced by HADS subscale scores above or just below the cut-offs, indicating anxiety and depressive symptoms. Participation restrictions were still evident and more pronounced compared to other studies, with an average USER-P restriction subscale score of 55 at 18 months, compared to scores above 80 in other studies after the first years after ABI [43,44,45]. Life satisfaction remained low among patients and family members, although this is not uncommon, even in the face of reduced caregiver burden [46].

Given the high emotional distress and the low level of participation, more intensive support may be needed. In our study, participants received on average 2.4 hours of CM support per month. In earlier studies on CM for PwABI [21] dose and frequency are not reported which impedes comparison. In the field of dementia there is a wide range in frequency of CM from once a week to once every 3 months and duration ranged from 4 to 12 months [16]. An important difference between people with ABI and dementia is the degenerative nature of the latter which requires increasing support over time.

Difficulties in finding and accessing appropriate support are known sources of burden and frustration for both PwABI and family members [8,47]. The process evaluation conducted alongside this trial showed that most participants were satisfied with their CMr support, finding comfort and relief in having someone to rely on to help navigate the healthcare system [48]. These positive experiences, combined with the reduction in unmet needs and caregiver burden, suggest that CM reduces some burden and stress associated with finding the right support.

However, the remaining stress and burden may reflect the persistent impact of ABI in major life activities and the constant need for adaptation to daily life events. CM for people with complex problems on multiple life domains has relevance within the health care system as no other service provides care coordination based on a holistic assessment of issues within the individual context, allowing frequent communication, and ongoing problem solving for PwABI and their family. Given that many people have difficulty accepting and emotionally processing the life-changing consequences of ABI [8,49], it is worth exploring whether psychotherapeutic approaches could further relieve psychosocial distress by facilitating constructive coping mechanisms and acceptance [50,51].

CM was offered in three different regions and by CMrs ith diverse backgrounds. Potentially these factores may have influenced outcomes. However, the regions were merely geographically different because the CM approach, CMr training and meetings were organized for all regions together. Moreover, the CMrs worked as teams with regular intercollegiate contact in order to complement eachothers background.

A few limitations should be mentioned. PwABI and family members were not involved in the study design, nor evaluation and interpretation of the results. Mostly due to the COVID-19 pandemic, recruitment and adherence were lower than expected. Due to the lack of a control group, it is unclear whether improvements can be attributed to CM specifically. In addition, the group of family members was small with limited power to detect differences in this group, which is also the case for the 24-month assessment for the PwABI. Not all PwABI had objectified ABI: this inclusion criterion was loosened after several intakes of people experiencing problems that were highly likely to be related to ABI (because of an injury/incident in the past) but for whom a formal diagnosis was not given at the time and for whom no other help was available. COVID-19 restrictions complicated CMr visits and study participation, and may be responsible for the increase in participation restrictions (according to the USER-P) for PwABI at 6 and 12 months.

## Conclusion

The results of this explorative study are cautiously promising and show some improvements in psychosocial outcomes in PwABI with problems on multiple life domains and their family members receiving CM. It is important to conduct further research on CM for this group, applying theories, models and framweorks from implementation science, but also to investigate how we can identify those who are at risk of getting stuck at an earlier moment and provide help more quickly.

## Additional File

The additional file for this article can be found as follows:

10.5334/ijic.8649.s1 Supplementary File. Supplementary Tables and Figure.
