# Supplementary material for: Case Management for People with Acquired Brain Injury with Complex Problems (Part 1): Outcomes of a One-group Trial
Source: Int J Integr Care. 2025 Jul 7;25(3):5. doi: 10.5334/ijic.8649 (PMC12247802; doi:10.5334/ijic.8649)

Supplementary Tables and Figure

Supplementary Table 1. Results for the secondary outcomes of PwABI for the longitudinal analysis from baseline to 18 months.

			Adjusted effects estimates		
	N	Observed Mean (SD)	Mean difference (95% CI)	P-value	Overall[a]
HADS anxiety					
baseline	62	9.16 (5.32)	reference		**F(3) = 2.772, p = .044**
6 months	43	8.16 (4.87)	-0.95 (-2.10-0.21)	.106	
12 months	44	7.77 (4.46)	-1.23 (-2.37- -0.08)	**.036***	
18 months	38	7.53 (5.09)	-1.62 (-2.82- -0.41)	**.009**	
HADS depression					
baseline	62	8.21 (4.73)	reference		F(3) = 1.019, p = .386
6 months	43	8.16 (4.52)	0.13 (-0.92-1.18)	.804	
12 months	44	8.20 (5.33)	0.15 (-0.90-1.19)	.783	
18 months	38	7.27 (4.90)	-0.74 (-1.84-0.36)	.183	
USER-P					
baseline	62	65.03 (22.93)	reference		**F(3) = 5.527, p = .001**
6 months	43	56.87 (21.25)	-7.52 (-11.66- -3.39)	**<.001**	
12 months	42	53.88 (24.85)	-10.00 (-15.49- -4.52)	**<.001**	
18 months	38	56.16 (25.39)	-6.93 (-13.49- -0.38)	**.038***	
LiSat					
baseline	62	33.84 (8.24)	reference		F(3) = 2.427, p = 0.69
6 months	43	32.26 (7.95)	-1.72 (-3.34- -0.10)	**.038***	
12 months	41	32.38 (9.48)	-1.44 (-3.59-0.71)	.188	
18 months	38	34.29 (9.32)	0.30 (-2.25-2.85)	.819	
PAM					
baseline	62	50.36 (10.05)	reference		F(3) = 0.849, p = .469
6 months	43	53.27 (11.85)	1.50 (-1.29-4.30)	.290	
12 months	43	52.69 (11.16)	2.05 (-0.75-4.84)	.150	
18 months	39	52.11 (11.49)	1.68 (-1.22-4.57)	.254	
LUNS					
baseline	62	7.09 (4.31)	reference		**F(3) = 15.059, p < .001**
6 months	43	5.21 (4.16)	-1.62 (-2.64- -0.60)	**.002**	
12 months	41	4.86 (5.07)	-2.41 (-3.44- -1.37)	**<.001**	
18 months	39	3.72 (4.49)	-3.39 (-4.44- -2.34)	**<.001**	

*no longer significant after applying Bonferroni correction. [a] Overall refers to the overall longitudinal trend.

Supplementary Table 2 Results for PAM level and observed number and percentage of PwABI per level for for the longitudinal analysis from baseline to 18 months.

	N	Level 1	Level 2	Level 3	Level 4	Overall[a]
baseline	62	30 (48.4)	14 (22.6)	16 (25.8)	2 (3.2)	F(3) = 1.658, p = 0.178
6 months	43	15 (34.9)	16 (37.2)	8 (18.6)	4 (9.3)	
12 months	43	13 (30.2)	14 (32.6)	13 (30.2)	3 (7.0)	
18 months	39	19 (48.7)	9 (23.1)	8 (20.5)	3 (7.7)	

[a] Overall refers to the overall longitudinal trend.

Supplementary Table 3. Results for the secondary outcomes of family members for for the longitudinal analysis from baseline to 18 months.

	N	Observed Mean (SD)	Adjusted effects estimates: Mean difference (95% CI)	P-value	Overall[a]
HADS anxiety					
baseline	36	9.56 (4.49)	reference		**F(3) = 4.823, p = .004**
6 months	25	8.00 (4.36)	-1.38 (-2.99-0.23)	.091	
12 months	24	6.96 (4.23)	-2.50 (-4.22- -0.96)	**.002**	
18 months	21	7.05 (4.34)	-2.75 (-4.46- -1.04)	**.002**	
HADS depression					
baseline	36	7.22 (4.78)	reference		F(3) = 1.240, p = .307
6 months	25	6.12 (4.79)	-1.21 (-2.61-0.20)	.091	
12 months	24	5.96 (4.42)	-1.31 (-2.92-0.31)	.110	
18 months	21	6.57 (5.24)	-1.02 (-3.03-0.99)	.309	
LiSat					
baseline	36	37.75 (7.86)	reference		F(3) = 0.729, p = .542
6 months	25	38.77 (8.23)	0.55 (-1.30-2.40)	.557	
12 months	24	37.42 (9.45)	-0.84 (-3.37-1.69)	.506	
18 months	21	37.44 (9.83)	-0.67 (-4.17-2.83)	.695	
CSES care management					
baseline	36	26.92 (9.26)	reference		F(3) = 1.057, p = .373
6 months	25	28.28 (10.31)	1.69 (-1.05-4.43)	.222	
12 months	24	28.83 (10.09)	1.96 (-0.82-4.74)	.164	
18 months	21	28.52 (10.14)	2.21 (-0.71-5.12)	.136	
CSES care use					
baseline	36	20.78 (7.34)	reference		**F(3) = 3.103, p = .032**
6 months	25	24.00 (6.87)	3.29 (1.04-5.55)	**.005**	
12 months	24	24.29 (6.36)	3.36 (0.51-6.20)	**.021***	
18 months	21	24.00 (6.64)	3.20 (-0.07-6.48)	.055	
CSI					
baseline	36	8.44 (2.98)	reference		**F(3) = 4.464, p = .006**
6 months	25	7.83 (3.11)	-0.45 (-1.10-0.19)	.166	
12 months	24	6.89 (3.52)	-1.15 (-1.81- -0.49)	**<.001**	
18 months	21	7.86 (3.21)	-0.80 (-1.49- -0.12)	**.023***	
FNQ community support					
baseline	36	4.80 (2.20)	reference		F(3) = 1.978, p = .126
6 months	25	4.77 (2.46)	-0.16 (-1.05-0.73)	.716	
12 months	23	6.08 (2.25)	0.90 (-0.18-1.98)	.101	
18 months	21	4.95 (1.99)	0.05 (-1.14-1.24)	.934	
FNQ emotional support					
baseline	36	6.40 (4.06)	reference		F(3) = 1.848, p = .147
6 months	25	8.11 (3.95)	1.64 (0.16-3.13)	**.030***	
12 months	23	8.21 (4.36)	1.66 (-0.17-3.48)	.074	
18 months	21	7.20 (3.84)	0.90 (-1.12-2.92)	.378	
FNQ health information					
baseline	36	11.47 (5.45)	reference		F(3) = 2.177, p = .099
6 months	25	13.60 (4.86)	1.98 (0.29-3.68)	**.023***	
12 months	23	13.70 (5.04)	1.86 (-0.34-4.07)	.096	

18 months	21	11.50 (6.43)	0.61 (-1.94-3.15)	.638	
FNQ instrumental support					
baseline	36	6.11 (3.08)	reference		F(3) = 0.235, p = .871
6 months	25	6.26 (2.95)	0.14 (-0.92-1.19)	.799	
12 months	23	6.90 (3.11)	0.48 (-0.85-1.80)	.478	
18 months	21	6.05 (3.20)	0.10 (-1.40-1.59)	.898	
FNQ involvement with care					
baseline	35	3.57 (1.82)	reference		F(3) = 0.886, p = .453
6 months	25	3.80 (1.53)	0.23 (-0.40-0.86)	.467	
12 months	23	3.91 (1.65)	0.33 (-0.46-1.12)	.405	
18 months	21	3.19 (1.83)	-0.23 (-1.12-0.66)	.609	
FNQ professional support					
baseline	36	4.26 (3.47)	reference		F(3) = 2.740, p = .051
6 months	25	5.92 (2.86)	1.63 (0.45-2.80)	**.008**	
12 months	23	6.00 (2.91)	1.45 (-0.003-2.91)	.051	
18 months	21	5.56 (3.01)	1.45 (-0.17-3.07)	.079	

*no longer significant after applying Bonferroni correction. [a] Overall refers to the overall longitudinal trend.

Supplmentary Figure 1. Mean number of hours invested by CMrs over 24 months.

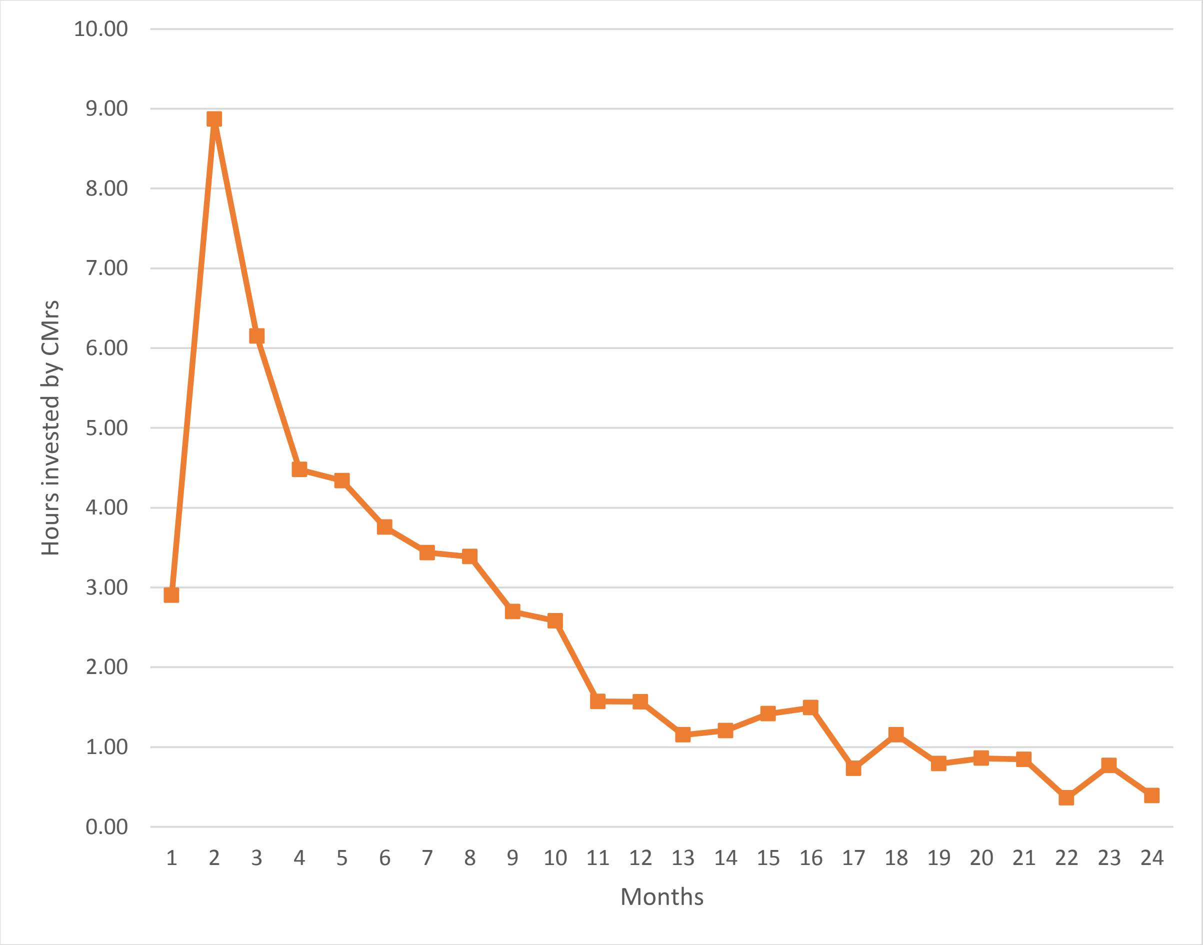

Supplement: Supplementary File. — Supplementary Tables and Figure. [file ijic-25-3-8649-s1.pdf]
